# Human Subcutaneous Dirofilariasis

**DOI:** 10.7759/cureus.35879

**Published:** 2023-03-07

**Authors:** Meghna Joseph, Mrinal Murali Krishna, Akhil Vijayan

**Affiliations:** 1 Pediatrics, Medical College Thiruvananthapuram, Thiruvananthapuram, IND; 2 Internal Medicine, Medical College Thiruvananthapuram, Thiruvananthapuram, IND; 3 Surgery, Smita Memorial Hospital and Research Centre, Thodupuzha, IND

**Keywords:** tropical infections, zoonotic diseases, subcutaneous dirofiariasis, mosquito-borne diseases, emerging infections, dirofilariasis

## Abstract

Dirofilariasis is a zoonotic infection transmitted by several species of mosquitoes. A 16-year-old boy presented with forearm swelling of two months duration. Imaging studies revealed a parasitic cyst. Surgical excision of the lesion was performed, and pharmacotherapy with diethylcarbamazine was given. A histopathological examination confirmed a diagnosis of human subcutaneous dirofilariasis caused by Dirofilaria repens. Clinicians should consider similar infections, especially in light of current climate changes and the emergence of various zoonoses. The epidemiological impact of diagnosing and preventing similar zoonotic infections is invaluable.

## Introduction

Human dirofilariasis is an emerging zoonotic infection, and it typically manifests in humans as pulmonary, ocular, or subcutaneous lesions [1]. This mosquito-borne infection occurs in humans as a result of aberrant inoculation of the microfilaria. We present a case of subcutaneous filariasis in a 16-year-old boy from Kerala, India. Human subcutaneous dirofilariasis is caused by Dirofilaria repens, a parasite in the subcutaneous tissue of dogs, cats, and other canids. It is transmitted by mosquitoes endemic to Southern and Eastern Europe and Asia, particularly Sri Lanka, Malaysia, and India [2]. The distribution pattern of human cases of subcutaneous dirofilariasis is similar to that of canine cases [2,3].

## Case presentation

**Figure 1:**
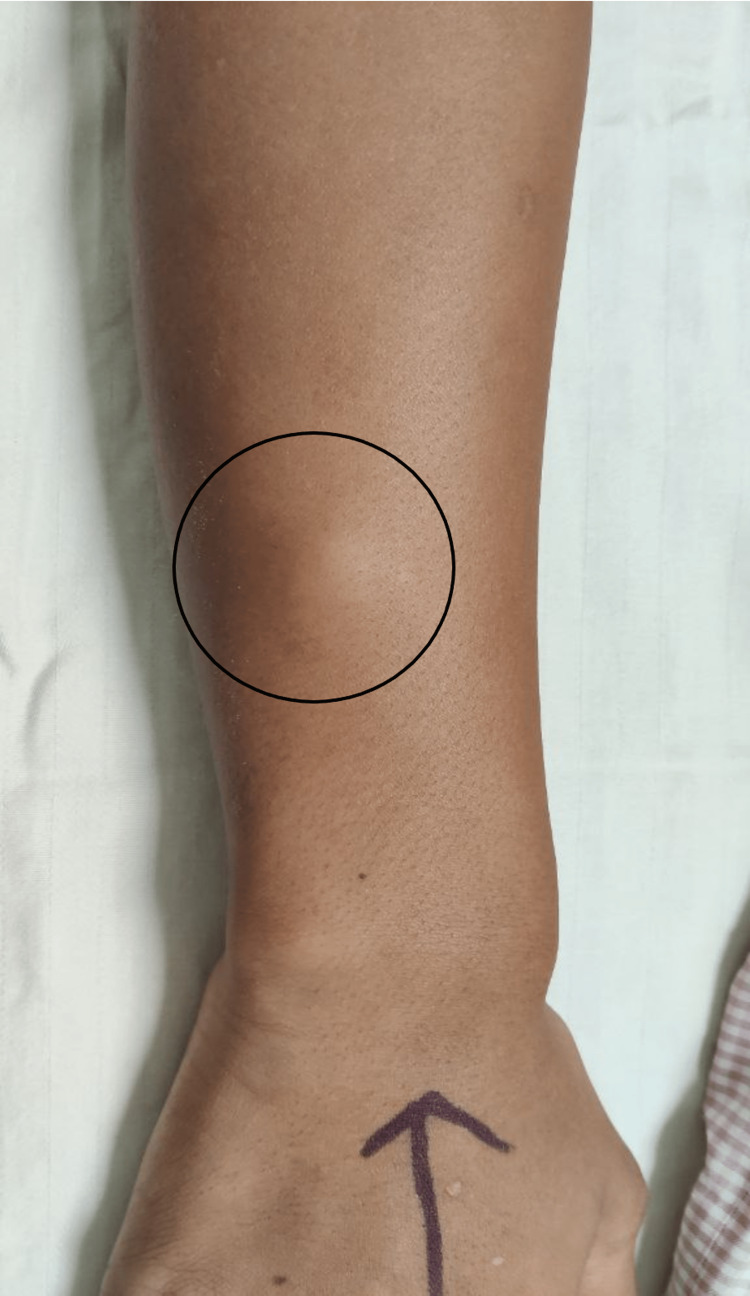
Swelling in the right forearm (black circle)

A 16-year-old boy, hailing from South India, presented with swelling in the right forearm for the past three months. The swelling was associated with mild pain. There was no associated itching, rash, cough, or abdominal symptoms. He had no significant past medical history. He had no history of contact with pets or recent travel. On clinical examination, a 3×2 centimeters (cm) sized well-defined firm swelling with restricted mobility was found on the dorsal aspect of the right forearm (Figure 1).

**Figure 2:**
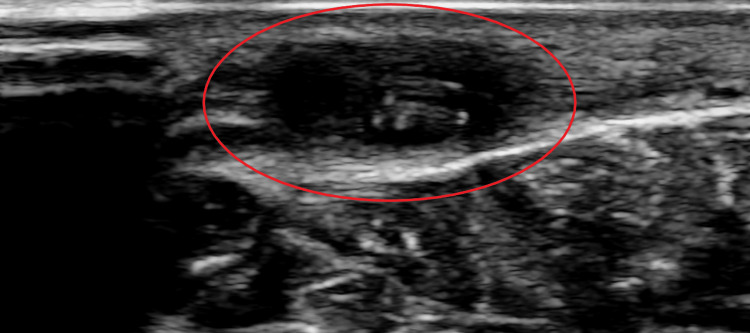
Ultrasonography Ultrasonography showing a subcutaneous hypoechoic lesion (red circle). Internal thin worm-like structures with parallel echogenic borders and anechoic centers can be seen.

There was no tenderness or local rise in temperature. Ultrasonography showed a 3×2 cm-sized hypoechoic lesion in the subcutaneous plane. Multiple internal thin worm-like structures with parallel echogenic borders and anechoic centers were seen (Figure 2).

**Figure 3:**
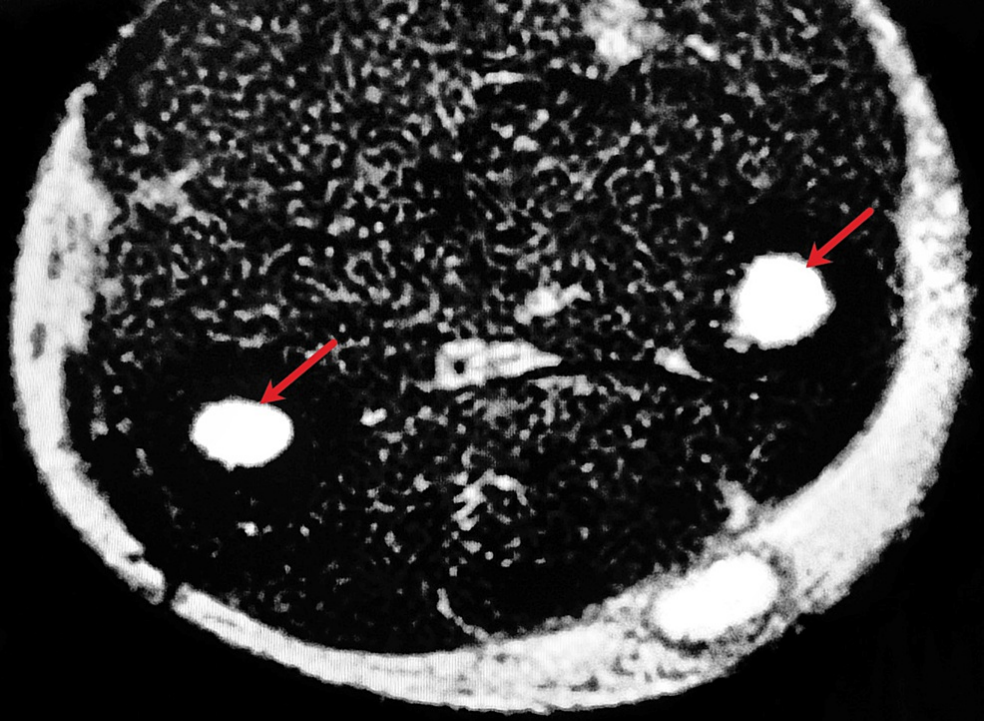
Magnetic resonance imaging A subcutaneous hyperintense circumscribed lesion with two structures (red arrows) within the lesion

These structures exhibited wriggling motion during real-time imaging. Magnetic resonance imaging (MRI) showed a hyperintense, subcutaneous, circumscribed lesion in the posteromedial aspect of the right forearm with a hypointense rim (Figure 3). A few hypointense curvilinear strands were seen within the lesion, with hyperintense edema around the lesion. Muscles, bones, and the rest of the soft tissue within the region appeared normal. MRI findings were suggestive of a parasitic cyst.

**Figure 4:**
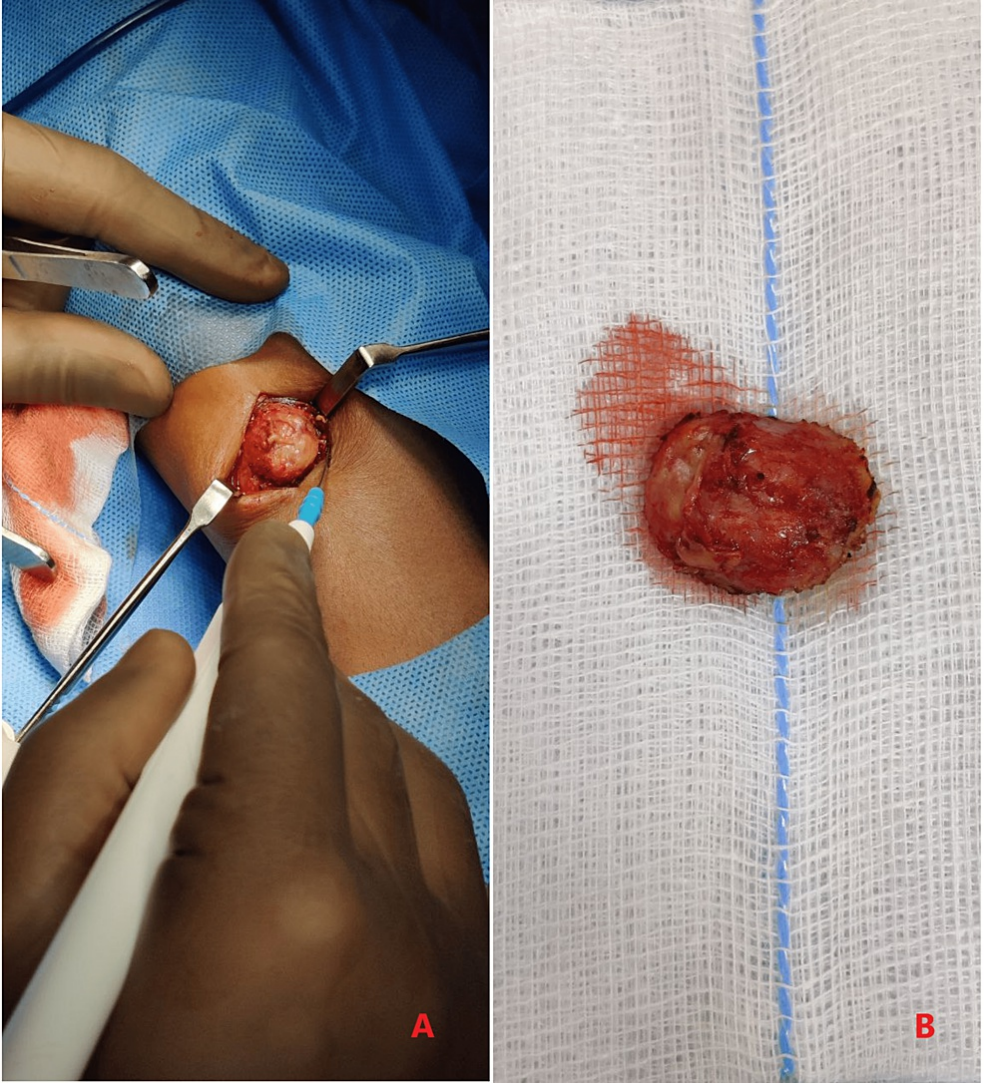
Gross appearance of the cyst Panel A shows an intraoperative image of a circumscribed cyst in the subcutaneous plane. Panel B shows the excised cyst.

**Figure 5:**
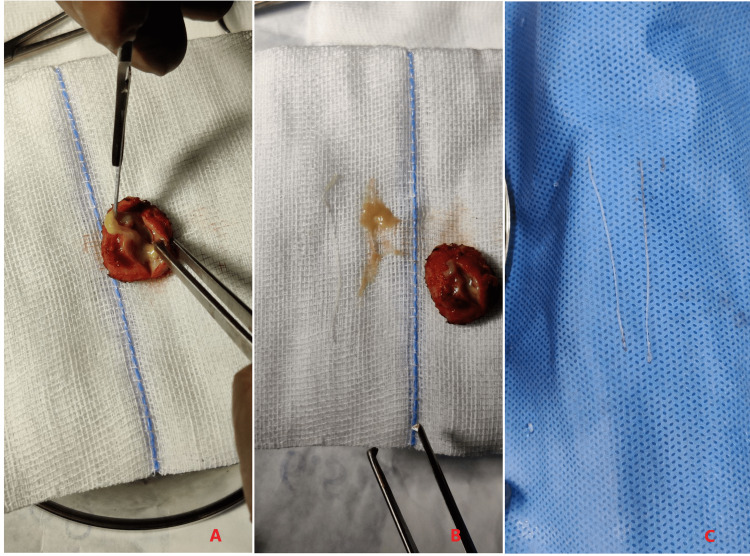
Contents of the cyst Panel A showing pus expelling from the cyst. Panel B shows pus and two worm-like structures obtained from the cyst. Panel C shows two 5 centimeters long worms.

Hemogram showed no eosinophilia and the peripheral blood smear was normal. Serum immunoglobulin (Ig)E level was normal. The swelling was excised in toto under local anesthesia (Figure 4). The contents of the swelling were pus and two 5 cm long slender tubular worm-like structures (Figure 5). The worms were identified to be adult female Dirofilaria.

**Figure 6:**
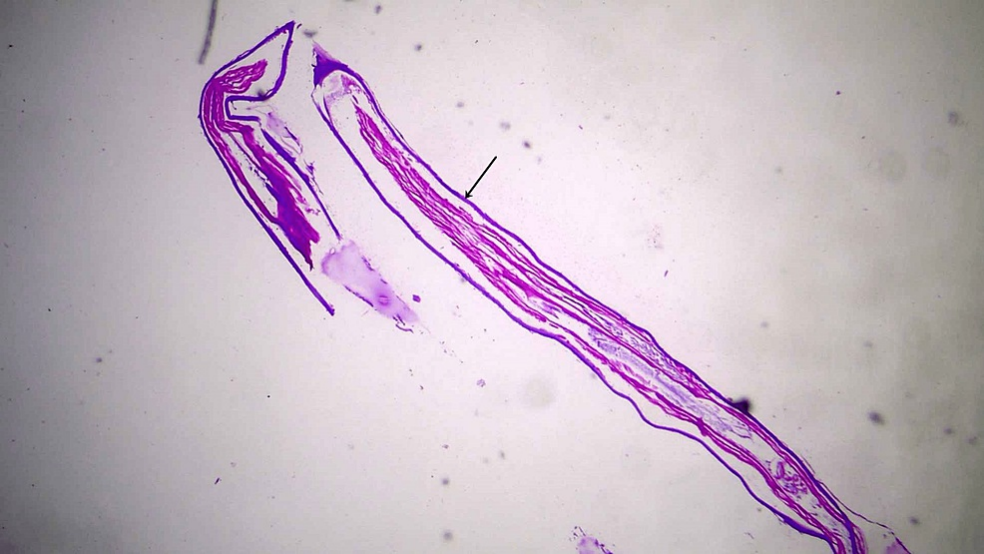
Histology image 1 Longitudinal section of the worm on hematoxylin and eosin staining showing thick cuticle (black arrow) and internal tubular structures.

**Figure 7:**
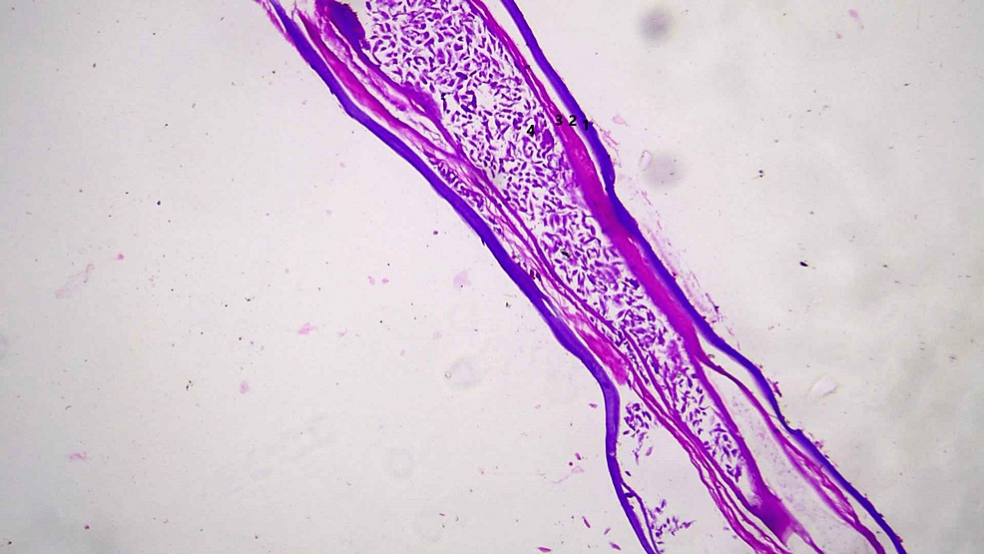
Histology image 2 Longitudinal section of the worm on hematoxylin and eosin staining showing multilayered cuticle (1), hypodermis (2), well-developed musculature (3), and reproductive tube with oocysts (4).

**Figure 8:**
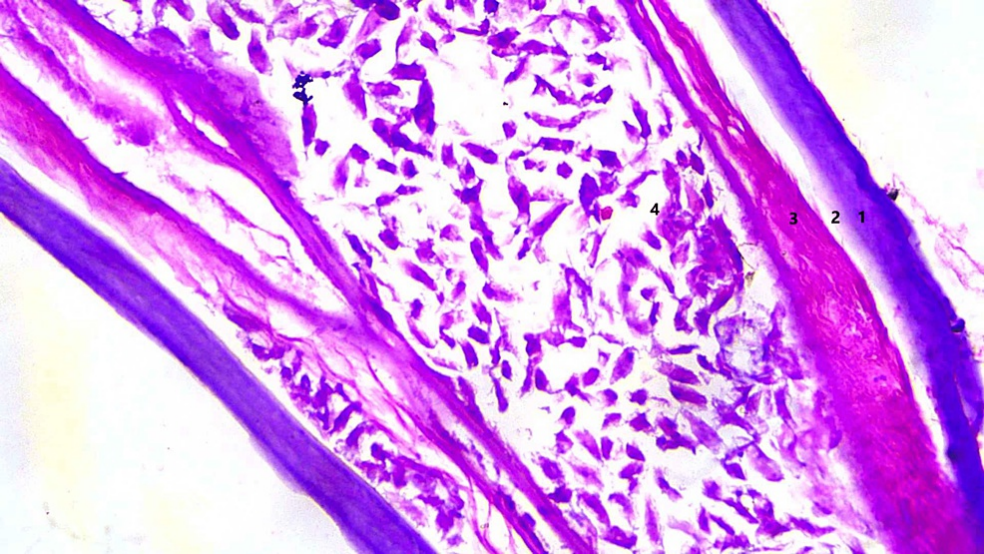
Histology image 3 Longitudinal section of the worm on hematoxylin and eosin staining at 100X magnification showing multilayered cuticle (1), hypodermis (2), well-developed musculature (3), and reproductive tube with oocysts (4).

Histopathologic examination of the cyst showed fibro-collagenous tissue with dense chronic inflammatory infiltrate composed of lymphocytes, epithelioid histiocytes, and eosinophils. Histologic examination of the worm-like structures showed a parasite with a thick multilayered cuticle with longitudinal ridges, prominent lateral cords, well-developed musculature, and ovaries with oocysts, consistent with female Dirofilaria (Figures 6-8). Thin and thick smear examinations did not show any evidence of microfilaremia.

## Discussion

Human dirofilariasis is a mosquito-borne zoonotic nematode infection. Dirofilaria immitis, D. repens and D. tenuis are the three most common Dirofilaria species causing human infections. Dogs and other wild canids are the natural hosts of these three Dirofilaria species. Transmission of the disease to humans occurs by bites of mosquito species like Aedes, Anopheles, Culex, and Mansonia [4]. D. immitis infection usually causes pulmonary lesions, often producing coin lesions that may or may not be symptomatic [5]. D.repens infection typically manifests as subcutaneous or submucosal nodules with adult worms inside [6]. 

The first-stage larvae circulating in the peripheral bloodstream of the natural hosts are taken up by mosquitos during a blood meal. The larvae mature inside the mosquito and the third-stage larvae are introduced to humans through a bite. Humans are accidental dead-end hosts of the nematode. The organisms mature into adults in humans and rarely cause microfilaremia. Like other filarial nematodes, D. repens hosts a bacterial endosymbiont, Wolbachia that has been known to manipulate the host immune response in these filarial infections [7,8].

Subcutaneous nodules caused by D. repens can arise anywhere in the body, predominantly affecting the upper body [4]. These usually present as migratory swellings with or without pain. Ocular dirofilariasis can be periorbital, subconjunctival, subtenon or intraocular. The differential diagnosis for subcutaneous dirofilariasis includes infected cysts or abscesses, lipomas, granulomas, and benign or malignant tumors. Peripheral blood eosinophilia and elevated IgE levels are not usually observed. Ultrasonography shows actively motile, tubular structures with parallel echogenic stripes within a cyst [9]. In our case, MRI showed a hyperintense subcutaneous lesion with hypointense curvilinear strands within the lesion, suggestive of a parasitic cyst. Histopathologic demonstration of Dirofilaria yields the definitive diagnosis. Dirofilaria can be identified by a multilayered thick cuticle with longitudinal ridges and well-developed circumferential musculature interrupted by lateral chords [10]. Histologic examination of the cyst often shows features of chronic inflammation. Microfilaremia is rarely seen in humans and there have been no reported cases of human-to-human transmission.

Surgical excision of the lesion is the definitive treatment for human subcutaneous dirofilariasis. Chemotherapy with ivermectin and diethylcarbamazine can be advised. In this patient, chemotherapy was given for five days considering the prevalence of vector mosquitoes in the region [11]. Avoidance of mosquito bites is the best method to prevent dirofilariasis. Eradication of Dirofilaria infection in dogs with combination therapy of ivermectin and doxycycline can be implemented to prevent potential public health issues [12]. Climate changes could be contributing to the increase in the incidence of similar zoonotic infections as such factors can affect mosquito breeding and disease transmission. An increase in temperature can shorten the lifecycle duration and generation time of various vector species, suitable for the potential transmission of disease-causing pathogens [13]. Changing climate can impact the land use patterns, which in turn can affect the habitat distribution of vector species promoting disease spread to newer areas and larger populations.

## Conclusions

Human subcutaneous dirofilariasis is an increasingly reported parasitic infection, especially in the tropical region. Prompt diagnosis is warranted as failure to diagnose can lead to unnecessary invasive investigations and procedures. Adequate measures like the use of insect-repellent creams or insecticide-treated bednets can be used to prevent mosquito bites.
